# Maladaptive reorganization of mediodorsal thalamus as a central mechanism in neuropathic pain-related sleep disorders

**DOI:** 10.1016/j.mmr.2026.100035

**Published:** 2026-05-07

**Authors:** Li Li, Qi Han, Hao-Lei Bai, Qin Xiao, Xie He, Jia-Fei Chen, Zhi-Ming Zhen, Xue-Qin Luo, Yuan-Jing Zhang, Min-Min Lu, Xiao Wang, Shi-Yin Li, Jia-Xiang Xiong, Yun Wang, Zhi-An Hu, Xiao-Long Zhang, Yong Liu, Chao He

**Affiliations:** Department of Pain and Rehabilitation, Xinqiao Hospital, Third Military Medical University, Chongqing 400038, China; 7 T Magnetic Resonance Imaging Translational Medical Center, Department of Radiology, Southwest Hospital, Third Military Medical University, Chongqing 400038, China; Department of Physiology, Third Military Medical University, Chongqing 400038, China; 7 T Magnetic Resonance Imaging Translational Medical Center, Department of Radiology, Southwest Hospital, Third Military Medical University, Chongqing 400038, China; Department of Pain and Rehabilitation, Xinqiao Hospital, Third Military Medical University, Chongqing 400038, China; Department of Physiology, Third Military Medical University, Chongqing 400038, China; Neuroscience Research Institute and Department of Neurobiology, School of Basic Medical Sciences, Key Laboratory for Neuroscience, Ministry of Education/National Health Commission and State Key Laboratory of Natural and Biomimetic Drugs, Peking University, Beijing 100083, China; Department of Physiology, Third Military Medical University, Chongqing 400038, China; Department of Pain and Rehabilitation, Xinqiao Hospital, Third Military Medical University, Chongqing 400038, China; Department of Physiology, Third Military Medical University, Chongqing 400038, China

**Keywords:** Neuropathic pain, Sleep disorders, Sleep fragmentation, Mediodorsal thalamus, Temporoparietal desynchronization

Dear Editor,

Chronic neuropathic pain and sleep disturbances exhibit high comorbidity, with bidirectional interactions forming a self-perpetuating “pain-sleep disturbance” vicious cycle that exacerbates clinical outcomes [1], [2]. Although evidence and mechanisms have been established linking poor sleep to the development of neuropathic pain [3], [4], the precise impact of neuropathic pain on sleep neurophysiology in clinical settings remains to be fully elucidated.

Zoster-associated neuralgia (ZAN), resulting from herpes zoster infection, represents a particularly severe clinical phenotype of neuropathic pain [5], [6]. Nevertheless, scant research has comprehensively examined the detailed characteristics of ZAN-associated sleep disturbances. Standardized questionnaires confirmed elevated pain severity [Neuropathic Pain ID Pain Scale (ID Pain), Visual Analogue Scale (VAS), Present Pain Intensity (PPI), and Numerical Rating Scale (NRS)] in 37 ZAN patients without pre-existing chronic insomnia, with subsequent sleep evaluations revealing a positive correlation between Pain Severity and Insomnia Severity Index (ISI) (**Additional file 1: Materials an d Methods; Table S1; Figs. S1, S2**).

Polysomnography (PSG) analysis revealed that ZAN patients manifested by an 51.30% increase in the number of awakenings (*P*=0.009), exhibited increased wake time after sleep onset (WASO) (*P*=0.004), when compared to healthy controls (HCs). The duration (*P*=0.003) and percentage (*P*=0.006) of rapid eye movement (REM) sleep and sleep efficiency (*P*=0.018) declined significantly, while the percentage (*P*=0.006) of non-rapid eye movement (NREM) sleep increased significantly. The total sleep time, sleep onset latency, duration of NREM sleep, and duration and percentage of each NREM sleep stage remained comparable to HCs (Fig. 1**a; Additional file 1: Table S2**). Transitional sleep patterns showed distinct instability, particularly in NREM stages: non-rapid eye movement sleep stage 2 (N2)-wakefulness (W)-non-rapid eye movement sleep stage 1 (N1) (*P*=0.025), N2-W-N2 (*P*=0.017), and non-rapid eye movement sleep stage 3 (N3)-N2-W (*P*=0.009) transitions occurred more frequently, while the number of other transition patterns did not change (**Additional file 1: Table S3**). The pain severity was positively correlated with these significant transition patterns (**Additional file 1: Fig. S3**). These results indicate that the sleep instability caused by ZAN primarily manifests as increased transitions from NREM sleep to wakefulness.

**Fig. 1:**
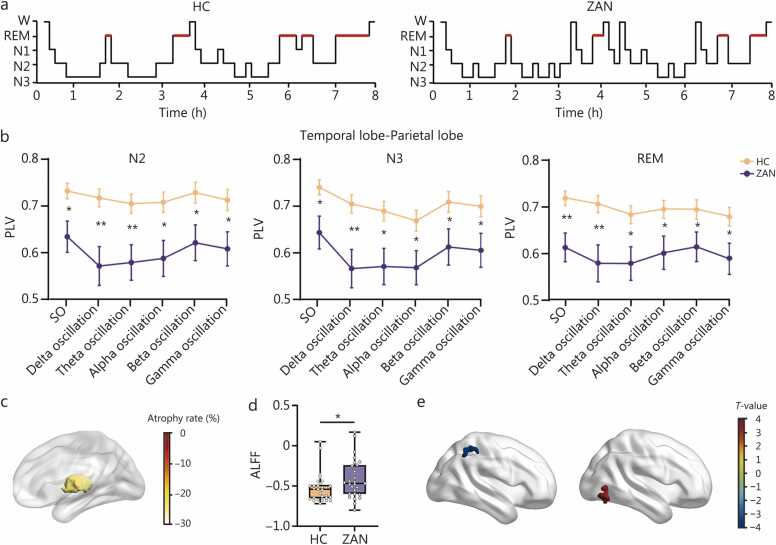
Maladaptive reorganization of mediodorsal thalamus may lead to temporal-parietal desynchronization and ZAN-associated sleep disturbance. **a** The representative hypnograms for HCs and ZAN patients. **b** PLV for different oscillations during N2, N3, and REM sleep. **c** The atrophy rate of the mediodorsal thalamus in ZAN patients. **d** The ALFF of mediodorsal thalamus differences in HCs and ZAN patients. **e** The alterations in the functional connections between mediodorsal thalamus and right inferior parietal lobe (left), right inferior temporal gyrus (right). Red regions represent significantly higher functional connections in ZAN patients, while blue indicates significantly lower. Group differences were analyzed using a two-sample t-test with age and sex as covariates, and a linear mixed model with subjects treated as a random effect and age and sex included as covariates. W. Wakefulness; N1. Non-rapid eye movement sleep stage 1; N2. Non-rapid eye movement sleep stage 2; N3. Non-rapid eye movement sleep stage 3; REM. Rapid eye movement; HC. Healthy controls; ZAN. Zoster-associated neuralgia; SO. Slow oscillation; PLV. Phase-locking value; ALFF. Amplitude of low-frequency fluctuations.;.

Next, the oscillatory network mechanisms underlying this sleep instability were explored. Despite the electroencephalogram spectral power didn’t show any significant difference across different brain lobes in distinct sleep stages when compared with HCs (**Additional file 1: Fig. S4**), phase synchrony between the temporal and parietal lobes showed a significant reduction predominantly observed during N2 and N3 stages of NREM sleep as well as during REM sleep stage (**Additional file 1: Table S4; Fig. S5a**), and manifested across slow, delta, theta, alpha, beta, and gamma oscillation (Fig. 1**b**). Functionally, the synchronous activity between the temporal lobe and parietal lobe was significantly negatively correlated with the transition patterns: N2-W-N1, N2-W-N2, and N3-N2-W. And decreased synchronicity during NREM sleep was positively correlated with the duration of REM sleep (**Additional file 1: Fig. S5b-d**), indicating that temporoparietal desynchronization may lead to the neuropathic pain-associated sleep instability.

Lastly, the structural and functional brain changes underlying temporoparietal desynchronization were investigated. Neuroimaging analysis revealed that ZAN patients exhibited a significant volume reduction in the mediodorsal thalamus (*P*<0.001; Fig. 1**c**) after family-wise error correction when compared to HCs. The mediodorsal thalamic atrophy was significantly correlated with the phase-locking value (PLV) between the temporal lobe and parietal lobe across various oscillations during N2, N3, and REM sleep stages, except for the gamma oscillation during N3 sleep, and the slow and delta oscillations during REM sleep (**Additional file 1: Table S5**). The atrophy of brain regions is always accompanied by changes in functional activity. Indeed, a significant increase in amplitude of low-frequency fluctuations was detected in ZAN patients (*P*=0.046; Fig. 1**d**). This result is consistent with previous reports showing that the dorsomedial thalamus plays a crucial role in both nociceptive signaling and sleep-wake dynamics [7] and its overactivation contributes to neuropathic pain development [8], suggesting that alterations in its functional activity and connectivity may contribute to pain-associated sleep disturbances. Additionally, the mediodorsal thalamus in ZAN patients exhibited significantly diminished functional connectivity with the right inferior parietal lobe (*T*=–3.686, *P*<0.001; Fig. 1**e**), while showing notably enhanced connectivity with the right inferior temporal gyrus (*T*=3.431, *P*<0.001; Fig. 1**e**). Of note, functional connectivity between mediodorsal thalamus and three other prefrontal regions including the right superior gyrus, right middle gyrus and right inferior gyrus was also decreased in ZAN patients (**Additional file 1: Fig. S6**).

Consistent with our human findings, rodent models of neuropathic pain demonstrate sleep fragmentation [9]. However, these rats exhibit selective NREM reduction without REM alterations, contrasting with the human ZAN phenotypes. This rodent-specific pathology associates with reticular thalamic nucleus hyperexcitability [9]. The differential sleep manifestations likely reflect distinct neuropathic pain etiologies (e.g., acute vs. chronic) and species-specific neural adaptations.

Targeted thalamic modulation represents a promising therapeutic strategy for pain-related sleep disorders. While a recent pilot study confirmed the safety of anterior cingulate cortex stimulation alone or combined with sensory thalamic stimulation, it demonstrated that isolated sensory thalamic stimulation failed to alleviate neuropathic pain [10]. Our findings revealed the possible mechanisms whereby neuropathic pain exacerbates sleep disturbances. The patients with ZAN experience a reduction of synchronization between the temporal and parietal lobes due to mediodorsal thalamus atrophy, and decreased functional connectivity between the mediodorsal thalamus and the prefrontal cortex. These complementary structural and functional thalamic alterations constitute a central pathophysiological axis underlying neuropathic pain-related sleep pathology. Thus, preventing dorsomedial thalamic degeneration or correcting its specific network dysregulation may constitute effective interventions for comorbid neuropathic pain and sleep disturbances.

## Abbreviations

HC: Healthy controls

ID Pain: Neuropathic Pain ID Pain Scale

ISI: Insomnia Severity Index

NRS: Numerical Rating ScaleN1: Non-rapid eye movement sleep stage 1

N2: Non-rapid eye movement sleep stage 2

N3: Non-rapid eye movement sleep stage 3

NREM: Non-rapid eye movement

PLV: Phase-locking value

PPI: Present Pain Intensity

PSG: Polysomnography

REM: Rapid eye movement

VAS: Visual Analogue Scale

W: Wakefulness

WASO: Wake time after sleep onset

ZAN: Zoster-associated neuralgia

## Ethics approval and consent to participate

This study was approved by the Ethics Committee of the Xinqiao Hospital of the Third Military Medical University of China (2021-Research No. 045-01). Written informed consent was obtained from all the participants. The diagnosis of ZAN was based on S2k guidelines for the diagnosis and treatment of herpes zoster and postherpetic neuralgia (2019 edition).

## Funding

This work was supported by the National Major Project of China Science and Technology Innovation 2030 for Brain Science and Brain-Inspired Technology (2021ZD0203201), the National Natural Science Foundation of China (32371038; U24A20373), and the Natural Science Foundation of Chongqing (2022NSCQ-JQX0019).

## Competing interests

The authors declared that they have no competing interests.

## Data Availability

All data generated or analyzed during this study are included in this published article and its supplementary information file. The raw structural MRI, functional MRI, and PSG data are publicly available on Open Neuro (https://openneuro.org/datasets/ds007181). All other data are available from the corresponding author upon reasonable request. Custom-made codes for two-sample *t*-test analysis, partial correlation analysis, linear mixed effect model analysis, spindles detection, PLV, and spectral power analyses are available on GitHub (https://github.com/ellebai/Publication-pain-and-sleep.git).
