# Supplementary material for: Maladaptive reorganization of mediodorsal thalamus as a central mechanism in neuropathic pain-related sleep disorders
Source: Mil Med Res. 2026 May 7;13(1):100035. doi: 10.1016/j.mmr.2026.100035 (PMC13186025; doi:10.1016/j.mmr.2026.100035)
Supplement: Supplementary file 1 — Supplementary material [file mmc1.pdf]

Materials and Methods

Participants

We performed a cross-sectional and observational study. A total of 44 patients with zoster-associated neuralgia (ZAN) were recruited from the Xinqiao Hospital, and 36 healthy controls (HC) were recruited from patients' families and nearby communities. The inclusion criteria were as follows: 1) meeting ZAN diagnostic criteria; 2) satisfied all protocol-defined inclusion requirements: baseline Numerical Rating Scale (NRS) score ≥4; 3) right-handed; 4) age 18–70 years; 5) ongoing pain in other hospitals; 6) no serious underlying disease; and 7) no pre-existing chronic insomnia verified through comprehensive medical history review, validated screening tools [e.g., Insomnia Severity Index (ISI)], and clinical assessment to confirm absence of chronic insomnia disorder (ongoing difficulties with initiating or maintaining sleep occurring at least three times per week, persisting for at least 3 months) prior to study enrollment. Exclusion criteria were as follows: 1) severe cardiac and pulmonary disease; 2) hematological disorders; 3) abnormal coagulation; 3) poor glycemic control; 4) malignancy; 5) use of immunosuppressants; and 6) magnetic resonance imaging (MRI) contraindications. The diagnosis of ZAN was based on S2k guidelines for the diagnosis and treatment of herpes zoster and postherpetic neuralgia (2019 edition) [1]. Specifically, study participants met the ZAN diagnostic criteria: a history of herpes zoster infection, the persistence of typical neuropathic pain rather than inflammatory pain following the resolution of the rash; described symptoms as lancinating, shooting, electrical-like, or stabbing sensations. The average duration from the onset of herpes zoster to study enrollment was (15.5±4.3) weeks.

In total, 7 ZAN patients and 2 HC were excluded due to incomplete questionnaires. Ten ZAN patients and 5 HC were excluded from the MRI analysis due to incomplete MRI data, excessive head movement (mean frame-wise displacement >0.2), or cerebrovascular events. Ten ZAN patients and 2 HC were excluded from polysomnography (PSG) analysis due to incomplete or unqualified data. Finally, 27 ZAN patients and 29 HC were included in the final MRI analysis, and 27 ZAN patients and 32 HC were included in the PSG analysis (**Additional file 1: Fig. S1**).

All participants underwent a comprehensive clinical and neuropsychological evaluation. Neuropathic Pain ID Pain Scale (ID Pain), Visual Analogue Scale (VAS), Present Pain Intensity (PPI), and NRS were used to assess pain severity. Sleep was assessed by the ISI. All scales mentioned above were administered by clinical physicians who have received specialized training in the assessment of these scales. This study was approved by the Ethics Committee of the Xinqiao Hospital of the Third Military Medical University of China (2021-Research, No. 045-01). Written informed consent was obtained from all the participants.

PSG data collection, preprocessing, and analysis

All participants were forbidden to consume beverages or food containing caffeine, tea, alcohol, or take any medicine that might affect sleep behaviors. PSG data were collected between 9 p.m. and 7 a.m. after electrodes were applied successfully with low impedance (<5 kΩ). Participants then completed a sleep diary to record their time in bed and wake time. To minimize the first-night effect, only data collected during the second night were used for subsequent analyses.

Electroencephalogram (EEG) signals were recorded by Ag/AgCl active electrodes mounted within an elastic cap, based on the 10–20 international electrode placement system. The signals were segmented into non-overlapping 30-second epochs, which were manually scored by experienced sleep technicians following the standards set by the American Academy of Sleep Medicine (AASM) [2]: 1) wakefulness shows low-voltage mixed-frequency EEG waves and alpha activity; 2) non-rapid eye movement sleep stage 1 (N1) sleep features theta waves (4–7 Hz) and slow eye movements; 3) non-rapid eye movement sleep stage 2 (N2) is identified by sleep spindles (11–16 Hz) and K-complexes; 4) non-rapid eye movement sleep stage 3 (N3) displays high-amplitude delta waves (0.5–2 Hz, >75 μV); 5) rapid eye movement (REM) sleep exhibits low-voltage mixed-frequency EEG resembling N1, coupled with rapid eye movements and sustained muscle atonia, particularly in the submental channel. The EEG signals consisted of 16 channels that were referenced to the contralateral mastoid. The electrooculogram (EOG) and electromyogram (EMG) were also collected simultaneously.

EEG signals were preprocessed using a standardized pipeline implemented in AutoMagic [3], including bad channel detection with the PREP pipeline, artifact correction (muscle, ocular, cardiac, etc.) using independent component analysis, line-noise removal with the ZapLine method, high-pass filtering at 0.1 Hz, and spherical interpolation of bad channels. Spectral power was then computed in EEGLAB (https://eeglab.org/), and phase-locking value (PLV) was estimated using HERMES [4] with default parameters across different sleep stages (wakefulness, N1, N2, N3 and REM sleep) and frequencies (slow oscillation: 0.5–1 Hz; delta: 1–4 Hz; theta: 4–8 Hz; alpha: 8–12 Hz; beta: 12–15 Hz; gamma: 15–30 Hz) to assess synchronization between channel pairs.

Standard PSG parameters, including the number of awakenings, total sleep time, wake time after sleep onset (WASO), sleep onset latency, sleep efficiency, and the duration and percentage of N1, N2, N3, REM, and non-rapid eye movement (NREM) sleep, were calculated based on sleep stage scoring. Transition patterns between sleep stages in ZAN patients versus HC were quantitatively analyzed using probability distributions of symbolic sequences. This novel analytical framework encompassed PSG transitions among all sleep stages (N1, N2, N3, REM) and wakefulness. The change rate was calculated based on the level in ZAN patients compared with HC using the following formula: (ZAN−HC)/HC.

MRI acquisition

MRI data were collected at 7 T Magnetic Resonance Imaging Translational Medical Center of Southwest Hospital with a Siemens Magnetom Terra 7 T MRI scanner (Siemens Healthineers, Erlangen, Germany), which was fitted with a 1 Tx and 32 Rx Nova Head Coil (Nova Medical, Wilmington, MA, USA). Participants were told to remain relaxed and awake, keeping their eyes closed during the scan. Foam pads were used to secure the subjects' heads to limit movement, and earplugs were employed to decrease noise exposure.

To start, the Fluid Attenuated Inversion Recovery (FLAIR) sequence scan was used to rule out any potential intracranial lesions, with the imaging parameters as follows: repetition time (TR)=9000 ms, inversion time (TI)=2600 ms, echo time (TE)=96 ms, slice thickness=3 mm, and scan time=3 min 9 s. Next, the Magnetization-Prepared with 2 Rapid Acquisition Gradient Echoes (MP2RAGE) sequence was collected using the parameters listed below: TR=4300 ms, TE=2.3 ms, slice thickness=0.65 mm, TI1=1000 ms, TI2=3200 ms, flip angle 1=4 degrees, flip angle 2=4 degrees, bandwidth=200 Hz/pixel, acceleration factor=3, and scan time=10 min 16 s. Subsequently, the resting-state functional magnetic resonance imaging (rs-fMRI) data were acquired utilizing an echo-planar imaging (EPI) sequence characterized by the following parameters: TR=2000 ms, TE=21.0 ms, slice thickness=1.5 mm, FA=90 degrees, bandwidth=1698 Hz/pixel, acceleration factor=2, voxel size=1.5 mm×1.5 mm×1.5 mm, slices=90, measurements=240, and scan time=8 min 16 s.

MRI preprocessing

Structural MRI was preprocessed using the Statistical Parametric Mapping (SPM) 12 (https://www.fil.ion.ucl.ac.uk/spm/software/spm12/) with the default settings. The preprocessing involved high-dimensional spatial normalization with a Dartel template in the Montreal Neurological Institute space, followed by nonlinear modulations and a correction for individual head size. Grey matter (GM) images (voxel size=1 mm×1 mm×1 mm) were obtained for group comparison. Participants with an interquartile range lower than 0.8 were excluded.

The preprocessing steps for functional MRI encompassed discarding the first four time points, realignment, normalization to the MNI echo-planar imaging (EPI) template (voxel size=1.5 mm×1.5 mm×1.5 mm), smoothing (full width at half maximum=3 mm×3 mm×3 mm), regressing out time series of white matter (WM 99% probability SPM map), cerebrospinal fluid (CSF 90% probability SPM map), and six head motion parameters. Subjects with a mean frame-wise displacement (FD) >0.5 mm were excluded. A 0.01–0.1 Hz band-pass filtering was also applied. The amplitude of low-frequency fluctuations (ALFF) was then calculated in a low-frequency band between 0.01 and 0.1 Hz to evaluate the local spontaneous activity and divided ALFF by the global mean for standardization [5].

Statistical analysis

Continuous variables were presented as mean±standard deviation (SD), and categorical variables were presented as proportions for each group. Group differences in demographic information, sleep structures, and PSG traits were assessed via two-sample *t*-tests (incorporating age and sex as covariates when appropriate) for continuous variables and chi-square tests for categorical variables. Group differences in oscillations at each stage were assessed using a linear mixed-effects model, with subjects modeled as a random effect and age and sex included as covariates. Group differences in GM volume were explored using a two-sample *t*-test model in SPM with age, sex, and total intracranial volume as covariates and corrected for multiple comparisons via family-wise error ($P<0.05$). The identified brain region showing significant differences in GM volume was set as the seed region to compute the functional connectivity with the whole brain using Pearson correlation. Group differences in ALFF and functional connectivity were explored using a two-sample *t*-test model in SPM with age, sex, and FD as covariates. Partial correlations among scales, sleep structures, PSG traits, and MRI metrics were performed with age, sex, and group as well as total intracranial volume and FD when appropriate as covariates, and multiple comparisons were corrected using false discovery rate (FDR) ($P<0.05$). The two-sample *t*-test analysis, partial correlation analysis, and linear mixed effect model were conducted using R-4.4.2.

References

1. Gross GE, Eisert L, Doerr HW, Fickenscher H, Knuf M, Maier P, *et al*. S2k guidelines for the diagnosis and treatment of herpes zoster and postherpetic neuralgia. JDDG J Dtsch Dermatol Ges. 2020;18(1):55-78.
2. Berry RB, Brooks R, Gamaldo C, Harding SM, Lloyd RM, Quan SF, *et al*. AASM Scoring Manual Updates for 2017 (Version 2.4). J Clin Sleep Med. 2017;13(5):665-6.
3. Pedroni A, Bahreini A, Langer N. Automagic: standardized preprocessing of big EEG data. NeuroImage. 2019;200:460-73.
4. Niso G, Bruña R, Pereda E, Gutiérrez R, Bajo R, Maestú F, *et al*. HERMES: towards an integrated toolbox to characterize functional and effective brain connectivity. Neuroinformatics. 2013;11(4):405-34.
5. Zang YF, He Y, Zhu CZ, Cao QJ, Sui MQ, Liang M, *et al*. Altered baseline brain activity in children with ADHD revealed by resting-state functional MRI. Brain Dev. 2007;29(2):83-91.

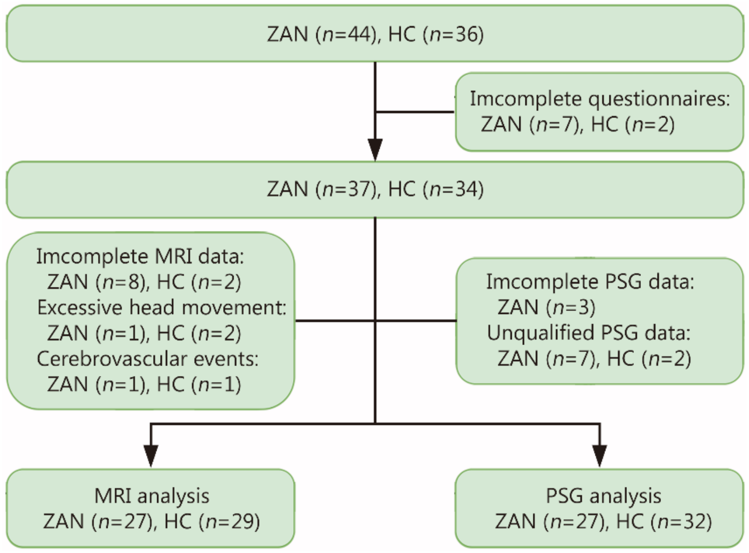

Fig. S1 Participant recruitment flowchart. HC. Healthy controls, ZAN. Zoster-associated neuralgia; MRI. Magnetic resonance imaging; PSG. Polysomnography

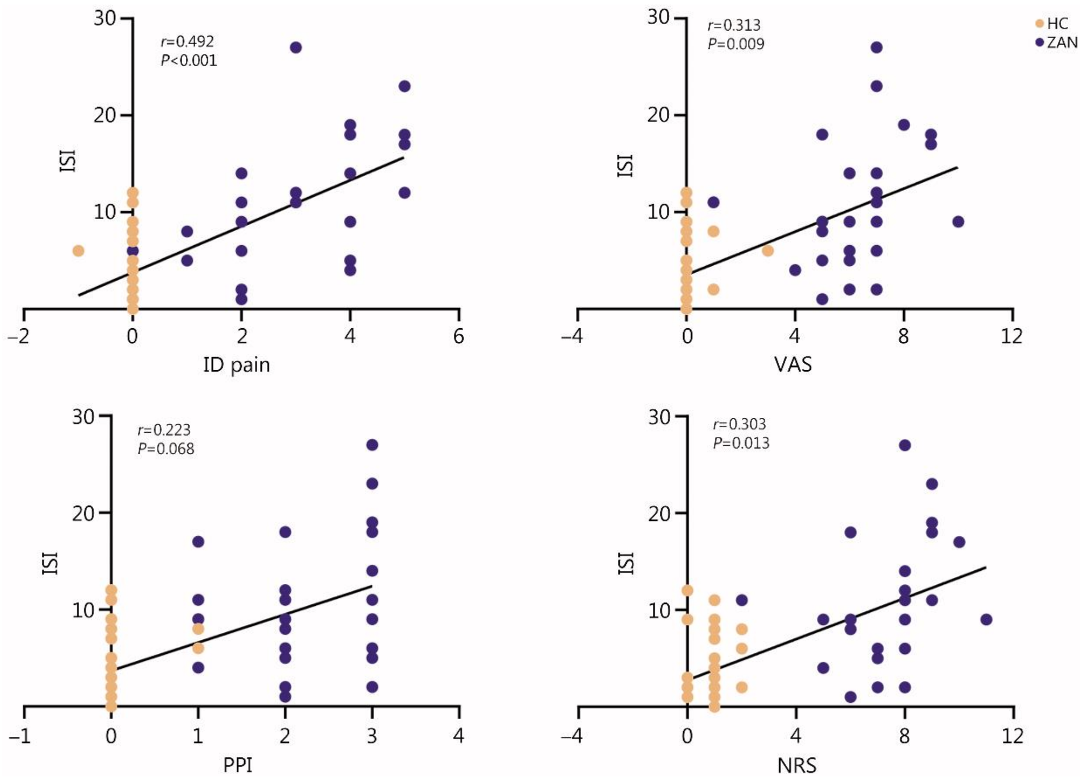

Fig. S2 Correlation between pain severity (ID pain, VAS, PPI, and NRS) and ISI. Partial correlation analysis was performed, controlling for age, sex, and group as covariates. ID Pain. Neuropathic Pain ID Pain Scale; VAS. Visual Analogue Scale; PPI. Present Pain Intensity; NRS. Numerical Rating Scale; ISI. Insomnia Severity Index; HC. Healthy controls; ZAN. Zoster-associated neuralgia

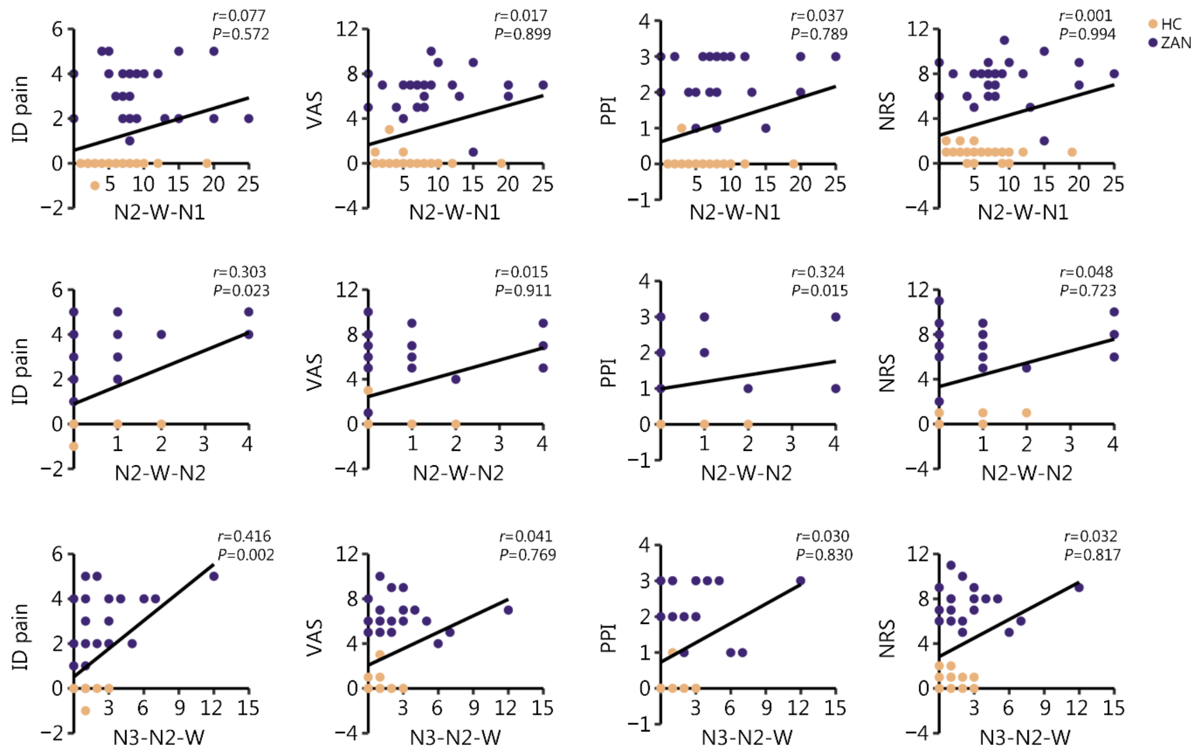

Fig. S3 Correlation between transition patterns (N2-W-N1, N2-W-N2, N3-N2-W) and pain severity (ID Pain, VAS, PPI, and NRS). Partial correlation analysis was performed, controlling for age, sex, and group as covariates. ID Pain. Neuropathic Pain ID Pain Scale; VAS. Visual Analogue Scale; PPI. Present Pain Intensity; NRS. Numerical Rating Scale; ISI. Insomnia Severity Index; W. Wakefulness; N1. Non-rapid eye movement sleep stage 1; N2. Non-rapid eye movement sleep stage 2; N3. Non-rapid eye movement sleep stage 3; HC. Healthy controls; ZAN. Zoster-associated neuralgia

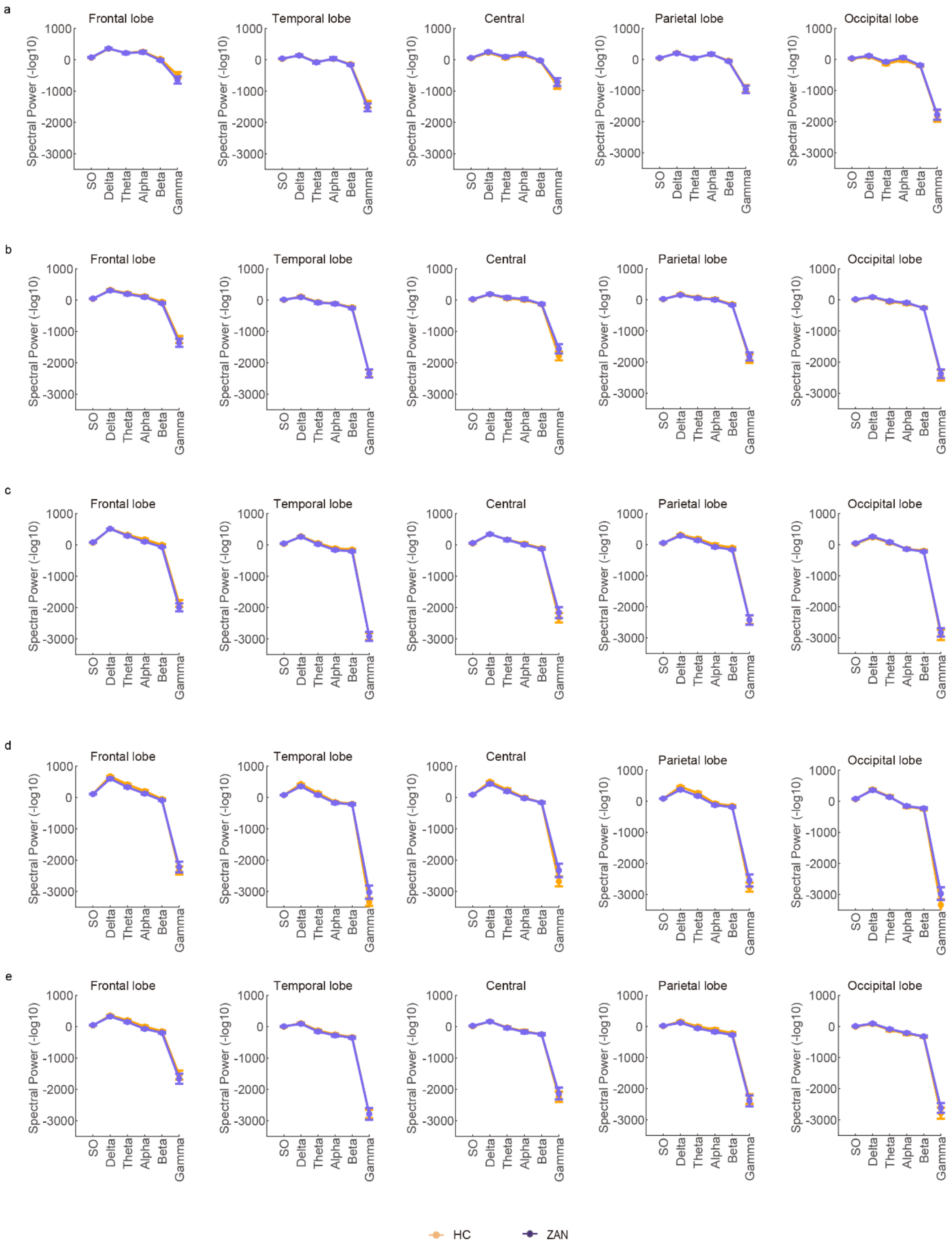

Fig. S4 Spectral power for different brain lobes in distinct sleep stages. The changes in the spectral power for different brain regions in W (**a**), N1 (**b**), N2 (**c**), N3 (**d**), and REM (**e**) sleep stages. Error bars represent the standard error of the mean. Group differences were analyzed using a linear mixed model, with subjects treated as a random effect and age and sex included as covariates. HC. Healthy controls; ZAN. Zoster-associated neuralgia; SO. Slow oscillation; REM. Rapid eye movement; W. Wakefulness; N1. Non-rapid eye movement sleep stage 1; N2. Non-rapid eye movement sleep stage 2; N3. Non-rapid eye movement sleep stage 3

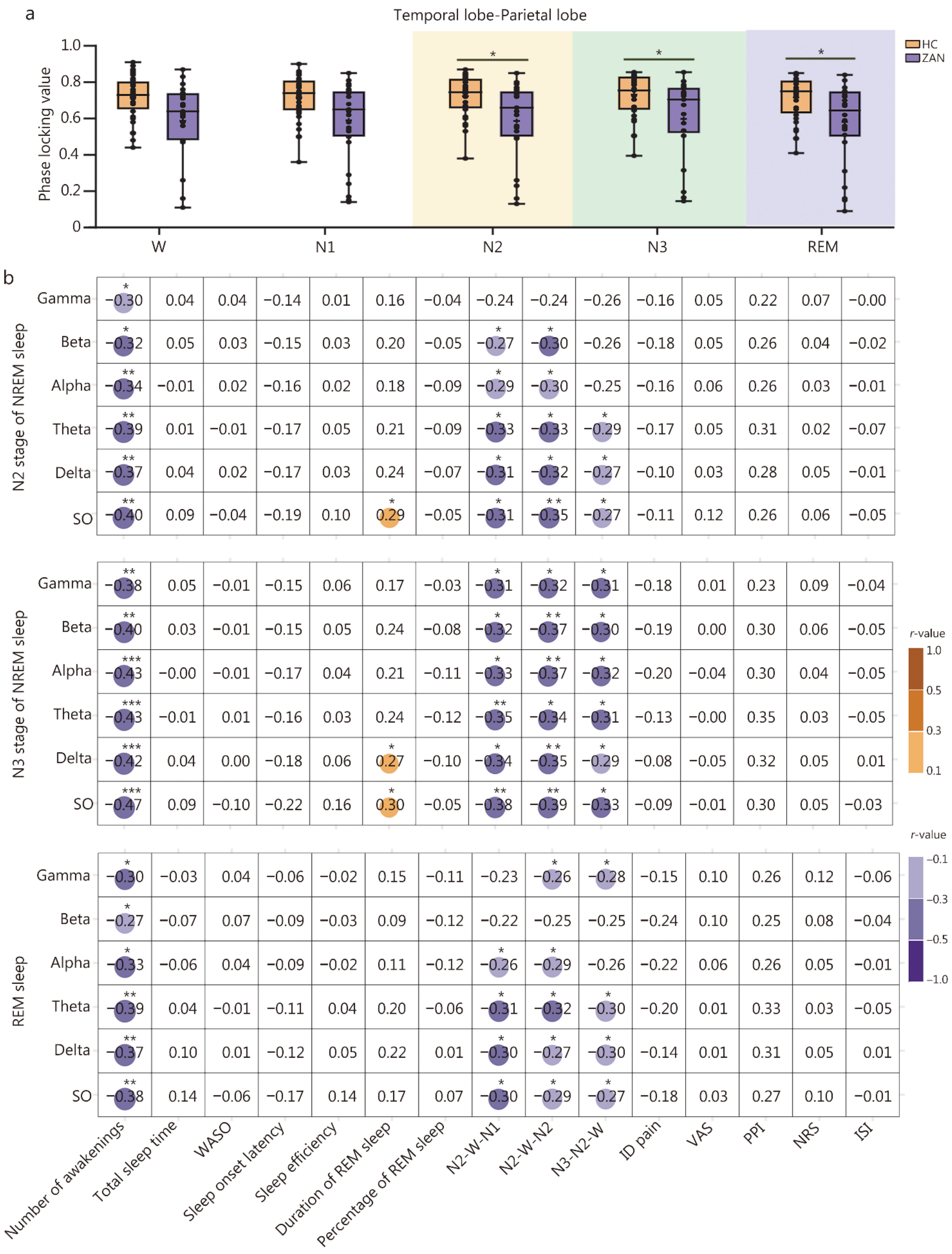

Fig. S5 The impaired temporoparietal synchronization was associated with both NREM sleep instability and REM sleep suppression. **a** Boxplots show the PLV for the whole frequency at different sleep and wakefulness stages. **b** The correlations between the synchronization strength of the temporal and parietal lobes, sleep structures (number of awakenings, total sleep time, WASO, sleep onset

latency, sleep efficiency, duration of REM sleep, percentage of REM sleep), and the pain severity (ID pain, VAS, PPI, NRS) during N2, N3, and REM sleep stages were examined. Colored dots represented significant correlations after being adjusted by FDR. Error bars represent the standard error of the mean. $^{*}P_{adj}<0.05$, $^{**}P_{adj}<0.01$. Group differences were analyzed using two-sample *t*-tests, with age and sex as covariates. Partial correlation analysis was performed, controlling for age, sex, and group as covariates. ID Pain. Neuropathic Pain ID Pain Scale; VAS. Visual Analogue Scale; PPI. Present Pain Intensity; NRS. Numerical Rating Scale; ISI. Insomnia Severity Index; REM. Rapid eye movement; N1. Non-rapid eye movement sleep stage 1; N2. Non-rapid eye movement sleep stage 2; N3. Non-rapid eye movement sleep stage 3; NREM. Non-rapid eye movement; W. Wakefulness; HC. Healthy controls; ZAN. Zoster-associated neuralgia; SO. Slow oscillation; PLV. Phase-locking value; WASO. Wake time after sleep onset

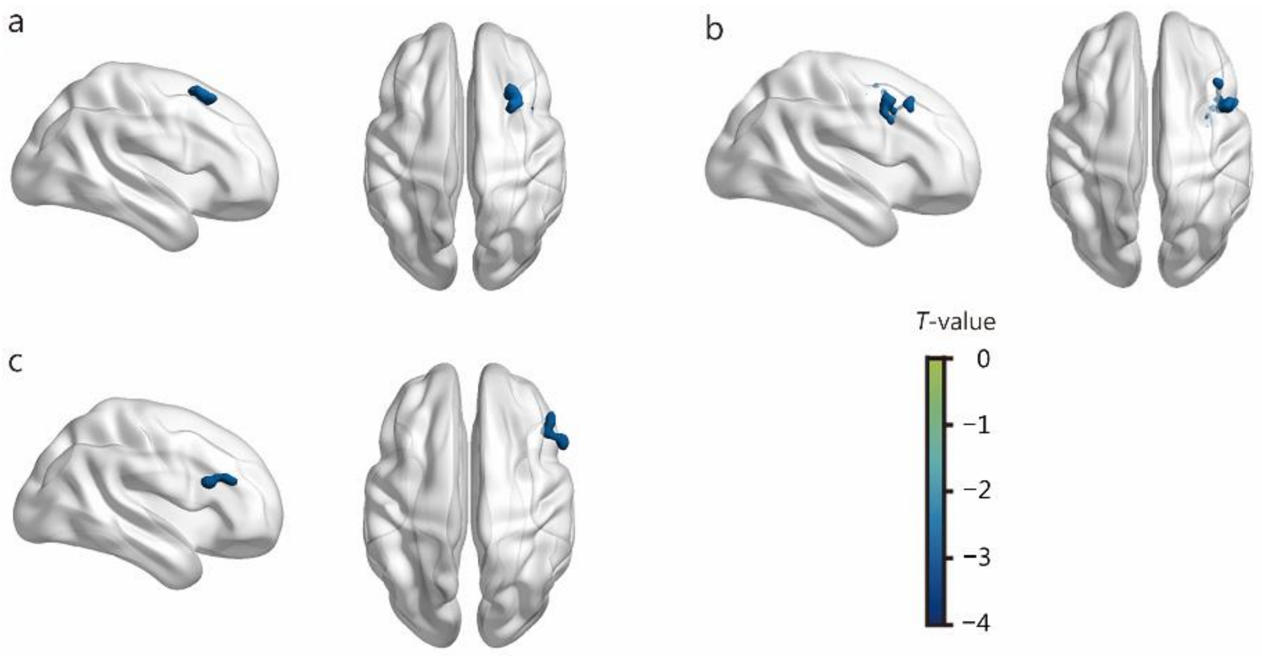

Fig. S6 Altered functional connections between the mediodorsal thalamus and the cortical regions. The alterations in the functional connections from mediodorsal thalamus to superior prefrontal gyrus (**a**), middle prefrontal gyrus (**b**), and inferior prefrontal gyrus (**c**). Blue indicates significantly lower. Group differences were analyzed using a two-sample *t*-test with age, sex and FD as covariates.

Table S1 Demographics and clinical characteristics of participants

Characteristics	HC (*n*=34)	ZAN (*n*=37)	*P*
Age [years, mean±SD]	61.176±4.668	61.865±7.142	0.630
Sex (Female/Male, *n*)	21/13	16/21	0.119
Education [*n* (%)]			0.201
Below middle school	1 (2.9)	3 (8.1)	
Middle school	7 (20.6)	13 (35.1)	
Higher than middle school	26 (76.5)	21 (56.8)	
ID pain (mean±SD)	−0.029±0.171	2.838±1.405	<0.001
VAS (mean±SD)	0.147±.558	6.514±1.835	<0.001
PPI (mean±SD)	0.058±0.239	2.351±0.789	<0.001
NRS (mean±SD)	0.912±0.514	7.541±1.865	<0.001
ISI (mean±SD)	3.676±3.364	11.324±6.988	<0.001
Disease duration (weeks, mean±SD)	/	15.541±4.336	-
Site of zoster onset (Left/Right, *n*)	/	19/18	-

/. No related records; -. No statistical results; SD. Standard deviation; ID Pain. Neuropathic Pain ID Pain Scale; VAS. Visual Analogue Scale; PPI. Present Pain Intensity; NRS. Numerical Rating Scale; ISI. Insomnia Severity Index; HC. Healthy controls; ZAN. Zoster-associated neuralgia

Table S2 The differences in sleep structures between HC and ZAN patients (mean±SD)

**Characteristics**	**HC**	**ZAN**	***P***
Number of awakenings	11.750±7.475	17.778±9.842	0.009
Total sleep time (min)	411.188±43.012	384.630±73.010	0.090
WASO (min)	37.594±28.730	68.926±52.930	0.004
Sleep onset latency (min)	12.031±14.987	9.722±14.975	0.560
Sleep efficiency (%)	0.891±0.073	0.829±0.121	0.018
Duration of REM sleep (min)	79.734±25.907	56.018±33.111	0.003
Duration of N1 sleep (min)	54.297±29.127	62.778±34.221	0.278
Duration of N2 sleep (min)	219.219±48.963	205.389±45.906	0.266
Duration of N3 sleep (min)	57.938±35.651	61.907±50.405	0.705
Duration of NREM sleep (min)	331.453±41.541	330.074±61.320	0.920
Percentage of REM sleep	0.193±0.059	0.142±0.081	0.006
Percentage of N1 sleep	0.132±0.069	0.171±0.102	0.061
Percentage of N2 sleep	0.532±0.099	0.535±0.094	0.901
Percentage of N3 sleep	0.143±0.089	0.151±0.110	0.723
Percentage of NREM sleep	0.807±0.059	0.858±0.081	0.006

SD. Standard deviation; WASO. Wake time after sleep onset; REM. Rapid eye movement; N1. Non-rapid eye movement sleep stage 1; N2. Non-rapid eye movement sleep stage 2; N3. Non-rapid eye movement sleep stage 3; NREM Non-rapid eye movement; HC. Healthy controls; ZAN. Zoster-associated neuralgia.

Table S3 The 25 most probable two-step transitions in HC and ZAN patients (mean±SD)

**Transition patterns**	**HC**	**ZAN**	***P***
N1			
N1-W-N1	5.531±8.780	7.926±6.120	0.237
N1-N2-W	10.125±8.292	12.222±8.768	0.345
N1-N2-N1	5.063±5.279	4.963±5.346	0.943
N1-N2-N3	4.406±2.487	5.630±4.869	0.225
N1-N2-REM	2.844±1.762	2.222±1.948	0.150
N1-REM-W	0.594±0.756	0.556±1.086	0.872
N1-REM-N1	1.781±2.028	1.370±1.843	0.410
N2			
N2-W-N1	6.219±3.782	9.111±5.866	0.025
N2-W-N2	0.218±0.490	0.814±1.272	0.017
N2-N1-W	0.750±0.984	0.926±2.218	0.691
N2-N1-N2	5.531±5.016	5.556±5.733	0.986
N2-N3-W	0.750±1.016	1.111±1.928	0.355
N2-N3-N2	19.500±8.791	29.074±30.146	0.087
N2-REM-W	1.031±1.062	1.259±1.483	0.476
N2-REM-N1	2.469±1.606	1.889±1.502	0.127
N2-REM-N2	0.844±1.139	0.667±0.920	0.503
N3			
N3-W-N1	0.719±0.991	0.880±1.509	0.617
N3-N2-W	1.156±1.110	2.600±2.692	0.009
N3-N2-N1	1.094±1.353	1.480±1.896	0.372
N3-N2-N3	16.125±8.075	25.960±28.001	0.057
N3-N2-REM	1.188±1.030	1.480±1.229	0.335
REM			
REM-W-N1	1.281±1.276	1.731±1.930	0.269
REM-N1-W	0.500±0.718	0.423±0.643	0.652
REM-N1-N2	2.125±1.476	1.846±1.592	0.477
REM-N1-REM	1.750±2.032	1.154±1.642	0.205

SD. Standard deviation; N1. Non-rapid eye movement sleep stage 1; N2. Non-rapid eye movement sleep stage 2; N3. Non-rapid eye movement sleep stage 3; REM. Rapid eye movement; HC. Healthy controls; ZAN. Zoster-associated neuralgia

Table S4 Phase-locking value (PLV) among different brain lobes for different sleep stages (mean±SD)

Regions	HC	ZAN	*P*	P_{adjust}
Wake				
Frontal lobe-Temporal lobe	0.451±0.128	0.435±0.132	0.782	0.839
Frontal lobe-Central area	0.590±0.217	0.527±0.173	0.234	0.655
Frontal lobe-Parietal lobe	0.271±0.138	0.252±0.116	0.566	0.707
Frontal lobe-Occipital lobe	0.282±0.138	0.265±0.106	0.459	0.655
Temporal lobe-Central area	0.541±0.216	0.491±0.168	0.371	0.655
Temporal lobe-Parietal lobe	0.717±0.118	0.587±0.225	0.007	0.066
Temporal lobe-Occipital lobe	0.503±0.250	0.459±0.209	0.425	0.655
Central area-Parietal lobe	0.525±0.191	0.634±0.141	0.018	0.091
Central area-Occipital lobe	0.273±0.183	0.323±0.167	0.381	0.655
Parietal lobe-Occipital lobe	0.597±0.250	0.588±0.184	0.839	0.839
N1				
Frontal lobe-Temporal lobe	0.422±0.138	0.407±0.133	0.842	0.842
Frontal lobe-Central area	0.583±0.221	0.521±0.172	0.266	0.532
Frontal lobe-Parietal lobe	0.221±0.137	0.202±0.114	0.634	0.720
Frontal lobe-Occipital lobe	0.301±0.134	0.259±0.104	0.155	0.517
Temporal lobe-Central area	0.538±0.219	0.497±0.159	0.454	0.720
Temporal lobe-Parietal lobe	0.716±0.120	0.589±0.214	0.007	0.064
Temporal lobe-Occipital lobe	0.486±0.255	0.408±0.223	0.214	0.532
Central area-Parietal lobe	0.505±0.183	0.621±0.148	0.013	0.064
Central area-Occipital lobe	0.254±0.186	0.286±0.155	0.557	0.720
Parietal lobe-Occipital lobe	0.593±0.260	0.565±0.193	0.648	0.720
N2				
Frontal lobe-Temporal lobe	0.433±0.143	0.414±0.132	0.697	0.774
Frontal lobe-Central area	0.590±0.219	0.533±0.176	0.277	0.497
Frontal lobe-Parietal lobe	0.212±0.127	0.174±0.116	0.228	0.497
Frontal lobe-Occipital lobe	0.311±0.128	0.286±0.113	0.298	0.497
Temporal lobe-Central area	0.546±0.227	0.500±0.167	0.414	0.591
Temporal lobe-Parietal lobe	0.723±0.112	0.586±0.217	0.003	0.030
Temporal lobe-Occipital lobe	0.467±0.262	0.366±0.236	0.128	0.425
Central area-Parietal lobe	0.502±0.175	0.585±0.167	0.075	0.377
Central area-Occipital lobe	0.247±0.185	0.260±0.147	0.873	0.873
Parietal lobe-Occipital lobe	0.587±0.268	0.555±0.192	0.613	0.767

**Regions**	**HC**	**ZAN**	***P***	P_{adjust}
N3				
Frontal lobe-Temporal lobe	0.440±0.149	0.425±0.141	0.812	0.902
Frontal lobe-Central area	0.587±0.220	0.544±0.188	0.465	0.717
Frontal lobe-Parietal lobe	0.205±0.118	0.174±0.108	0.344	0.687
Frontal lobe-Occipital lobe	0.326±0.136	0.299±0.114	0.318	0.687
Temporal lobe-Central area	0.532±0.226	0.499±0.167	0.573	0.717
Temporal lobe-Parietal lobe	0.716±0.113	0.583±0.224	0.005	0.047
Temporal lobe-Occipital lobe	0.439±0.263	0.361±0.246	0.224	0.687
Central area-Parietal lobe	0.481±0.172	0.569±0.176	0.083	0.413
Central area-Occipital lobe	0.238±0.170	0.247±0.132	0.918	0.918
Parietal lobe-Occipital lobe	0.574±0.273	0.540±0.195	0.572	0.717
REM				
Frontal lobe-Temporal lobe	0.404±0.151	0.412±0.146	0.737	0.737
Frontal lobe-Central area	0.583±0.223	0.527±0.182	0.340	0.679
Frontal lobe-Parietal lobe	0.206±0.134	0.177±0.121	0.428	0.714
Frontal lobe-Occipital lobe	0.336±0.143	0.303±0.115	0.268	0.670
Temporal lobe-Central area	0.529±0.215	0.500±0.147	0.602	0.737
Temporal lobe-Parietal lobe	0.716±0.114	0.581±0.222	0.004	0.044
Temporal lobe-Occipital lobe	0.472±0.268	0.352±0.232	0.083	0.277
Central area-Parietal lobe	0.483±0.176	0.591±0.156	0.019	0.097
Central area-Occipital lobe	0.236±0.178	0.270±0.146	0.542	0.737
Parietal lobe-Occipital lobe	0.588±0.260	0.563±0.181	0.680	0.737

SD. Standard deviation; N1. Non-rapid eye movement sleep stage 1; N2. Non-rapid eye movement sleep stage 2; N3. Non-rapid eye movement sleep stage 3; REM. Rapid eye movement; HC. Healthy controls; ZAN. Zoster-associated neuralgia

Table S5 Correlation between atrophy rate of mediodorsal thalamus and PLV between temporal and parietal lobes

**Oscillations**	**Atrophy rate of mediodorsal thalamus**	
	r	***P***
PLV during N2		
SO	−0.584	0.028
Delta	−0.600	0.023
Alpha	−0.662	0.010
Theta	−0.641	0.014
Beta	−0.636	0.015
Gamma	−0.556	0.039
PLV during N3		
SO	−0.605	0.029
Delta	−0.604	0.029
Alpha	−0.630	0.021
Theta	−0.611	0.027
Beta	−0.656	0.015
Gamma	−0.549	0.052
PLV during REM		
SO	−0.300	0.319
Delta	−0.541	0.056
Alpha	−0.698	0.008
Theta	−0.631	0.021
Beta	−0.648	0.017
Gamma	−0.654	0.015

PLV. Phase-locking value; N2. Non-rapid eye movement sleep stage 2; N3. Non-rapid eye movement sleep stage 3; REM. Rapid eye movement; SO. Slow oscillation
